# Effect of Grazing Behavior on Weight Regain Post-Bariatric Surgery: A Systematic Review

**DOI:** 10.3390/nu9121322

**Published:** 2017-12-05

**Authors:** Nathalia Pizato, Patrícia B. Botelho, Vivian S. S. Gonçalves, Eliane S. Dutra, Kênia M. B. de Carvalho

**Affiliations:** 1 Graduate Program of Human Nutrition, University of Brasília, Brasília 70910-900, Brazil; nathaliapizato@gmail.com (N.P.); vinut.bsb@gmail.com (V.S.S.G.); eliane.unb@gmail.com (E.S.D.); 2 Department of Nutrition, University of Brasilia, Brasília 70910-900, Brazil; patriciaborges.nutri@gmail.com

**Keywords:** grazing, eating behavior, weight regain, bariatric surgery, systematic review

## Abstract

Grazing, a type of maladaptive eating behavior, has been associated with poor weight outcomes in bariatric patients. The purpose of this study was to conduct a systematic review of the association between grazing behavior and weight regain post-bariatric surgery. Literature searches, study selection, design of the method, and quality appraisal were carried out by two independent authors. The search strategy was performed until October 2017 in Medline, Embase, Cochrane, Lilacs, Scopus, Web of Science, Google Scholar, ProQuest Dissertation & Theses, and Open Grey. Of a total of 3764 articles, five papers met the inclusion criteria (four original articles and one thesis), comprising 994 subjects, mostly women. The prevalence of grazing behavior ranged from 16.6 to 46.6%, and the highest prevalence of significant weight regain was 47%. The association between grazing and weight regain was observed in four of the five evaluated studies. Our findings support an association between grazing behavior and weight regain after bariatric surgery, regardless of surgery type and contextual concept of grazing. Further studies are needed to confirm the clarity of the real prevalence and interfering factors related to grazing behavior and weight outcomes.

## 1. Introduction

Bariatric surgery is considered the most effective treatment for severe obesity [1,2]. However, the presence of maladaptive eating behaviors may increase the risk of long-term postoperative weight regain [3,4]. The magnitude of weight regain depends on postoperative time and the type of bariatric surgery, with no consensus on the cutoff point to consider it clinically significant [5]. This weight regain may represent not only a frustration to patients [6], but also lead to a lack of control of comorbidities associated with obesity [7].

The identification and treatment of possible eating disorders or even a maladaptive eating behavior may be a key strategy for the management of bariatric patients [8,9]. Among the different types of maladaptive eating behaviors, grazing behavior draws attention because this condition might impact on treatment outcomes [10]. Even so, it is possible that researchers and health professionals in this field are neglecting or sub-reporting this behavior, since so far no strict diagnosis has been defined [11]. Similar behaviors—picking and nibbling (P&N), loss of control (LOC), or snack eating behavior [12]—may misreport the presence of grazing. Conceição et al. [11] brought this issue to light, proposing a unique definition of grazing as an eating behavior characterized by the repetitive eating of small amounts of food in an unplanned manner. 

Although previous studies have suggested the association between grazing behavior and weight regain as a plausible hypothesis [13,14], no systematic analysis in this field has been conducted in order to assess the level of scientific evidence of existing studies or support the planning of future protocols. Therefore, this systematic review aims to synthesize the best available evidence on the effect of grazing behavior on weight regain after bariatric surgery.

## 2. Materials and Methods 

This systematic review followed the Preferred Reporting Items for Systematic Reviews and Meta-analyses PRISMA checklist [15].

### 2.1. Protocol and Registration

The systematic review protocol was registered in the International Prospective Register of Systematic Reviews (PROSPERO), under registration number CRD42017071710 at 8 August 2017.

### 2.2. Eligibility Criteria

The present study included clinical trial and observational studies. Only studies with patients that have undergone any type of bariatric surgery and have analyzed the grazing behavior or picking & nibbling [11], and its effect on weight regain in the postoperative follow-up, were considered for retention.

The exclusion criteria were as follows: (1) procedures for weight loss other than bariatric surgery; (2) patients with mental disorders or other conditions that affect body weight, such as pregnancy, endocrine, and metabolic disorders; (3) reviews, abstracts, letters, personal opinions, and books.

In order to reduce publication and retrieval bias, the search was not restricted by language, publication date, or status.

### 2.3. Information Sources

The search strategy was elaborated according to the criteria established by the Peer Review of Electronic Search Strategies (PRESS checklist) [16], being reviewed by two researchers experienced in systematic reviews, and the suggested adequacies were incorporated.

Detailed individual search strategies for each of the following databases were conducted: Medline, Embase, Cochrane, Lilacs, Scopus, and Web of Science. A partial grey literature search was also performed in Google Scholar, ProQuest Dissertation & Theses, and Open Grey. A Google Scholar search was limited to the first 200 most relevant articles. The search was conducted on 9 July 2017 and updated on 19 October 2017. Additional articles, as indicated in the reference sections of papers, were searched by hand.

Appropriate word combinations were selected and adapted for each database search. More information on the search strategies is provided in Appendix A. Covidence Software (Cochrane Collaboration software^®^, Melbourne, Australia) was used to remove duplicate references and for the screening procedure, applied independently.

### 2.4. Study Selection 

The study selection process was carried out in two phases by two independent investigators (N.P. and P.B.B.). In phase 1, articles were selected based on their titles and abstracts. Articles that did not appear to meet the inclusion criteria were discarded. The remaining records were read in their entireties in phase 2, and those suitable for the review were selected. In cases where a consensus could not be reached by the two authors, a third author (E.S.D.) helped make a decision regarding the paper selection.

One examiner (N.P.) critically assessed the reference list of selected studies. Authors K.M.B.d.C and V.S.S.G. performed a final verification of the list of articles included on the basis of the eligibility criteria.

### 2.5. Data Collection Process

One author (N.P.) extracted data from the selected studies. A second author (P.B.B.) crosschecked all the information. Any disagreements between the two authors were discussed until resolved. A third author (E.S.D.) made the final decision when the two authors failed to reach an agreement.

Information on the following data items was recorded from the selected studies: authors, publication year, aim of study, study design/follow-up period, type of surgery, sample size, grazing behavior evaluative instrument, weight regain cut-off, weight regain prevalence, grazing behavior prevalence/status, and main result of grazing and weight regain correlation.

### 2.6. Risk of Bias within Individual Studies 

The critical appraisal tool, recommended by The Joanna Briggs Institute [17] for cross sectional studies, was used to assess their risk of bias. Two reviewers (N.P. and P.B.B.) independently assessed the quality of each study. A third reviewer (K.M.B.d.C.) resolved disagreements between the two main reviewers. The tool consisted of eight questions answered as “yes”, “no”, “unclear”, or “not applicable”. For this study, when all items were answered “yes”, the risk of bias was low and if any item was classified as “no”, a high risk of bias was expected. No scores were assigned; results were expressed by the frequency of each classification of the evaluation parameters. These ratings were not used as a criterion for study eligibility.

## 3. Results

### 3.1. Literature Search

**Figure 1:**
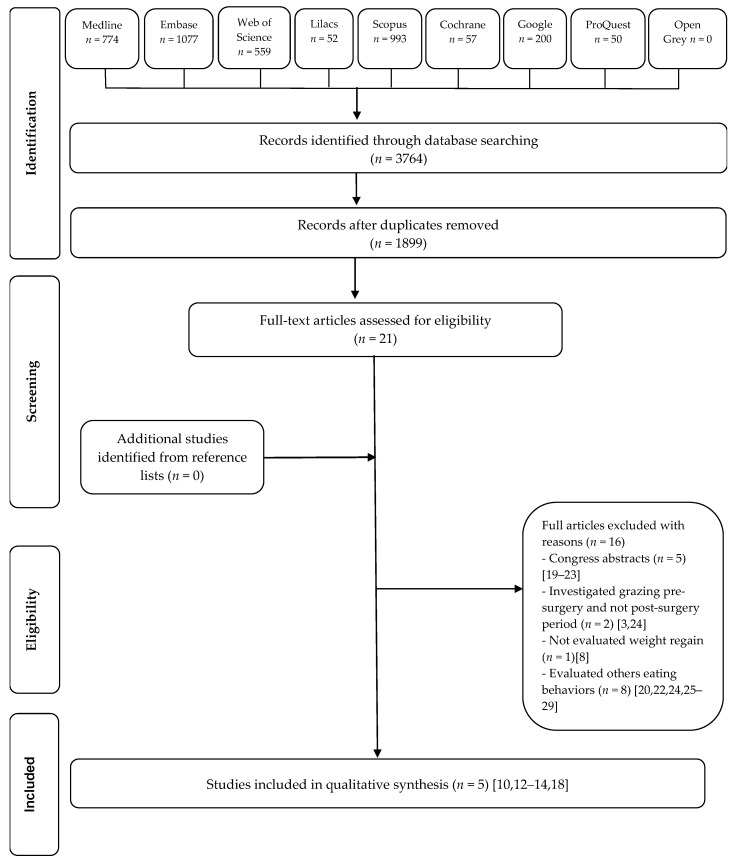
Flow diagram of the literature search and selection criteria.

A total of 3764 manuscripts were initially retrieved from the databases search. After duplicate removal, the titles and abstracts of 1899 articles were screened and 21 potential studies were assessed for full-text reading. Sixteen studies were excluded. No studies were identified by manual search from the reference list of the fully read articles. At the end, four original articles [10,12,13,14] and one thesis [18] were considered for this systematic review. Figure 1 shows a flow diagram of the screening process. 

### 3.2. Study Characteristics

Table 1 shows a summary of the study characteristics. The total sample size was 994 subjects, majority women, and the follow-up period range was from 6 months [12] to 10 years [13] post-bariatric surgery. The studies had a cross-sectional design, from Portugal [10,12], Spain [14], and the United States of America [13,18], and all of them evaluated patients who underwent Roux-en-Y Gastric Bypass (RYGB). Three of four studies also evaluated Sleeve Gastrectomy (SG) [10,14,18] and the other two included Laparoscopic Adjustable Gastric Banding (LAGB) [12,18]. Weight regain was defined as any increase from the lowest postsurgical weight [13], or when excessive weight regain was more than 10% [14] or 15% [12]. In two studies, the authors did not define the weight regain [10,18].

The authors used different definitions to evaluate grazing behavior. Conceição et al. [10,12] considered picking or nibbling behavior, as “eating modest amounts of food in an unplanned and repetitious way, without a sense of loss of control”, previously described by Fairburn et al. [30], while Nicolau et al. [14] applied grazing as “the consumption of small amounts of food continuously over an extended period of time, resulting in eating more than the subjects considers best for them”. Simpson et al. [18] considered “unplanned, continuous and repetitive eating of small amounts of food through extended time periods, associated to loss of control over eating”. In turn, Kofman et al. [13] used the following definition of grazing: “a pattern of eating or nibbling continuously at least 2 days a week for a 6-month period over an extended period of time in addition to an inability to stop or control their eating while nibbling”.

### 3.3. Risk of Bias within Individual Studies

**Figure 2:**
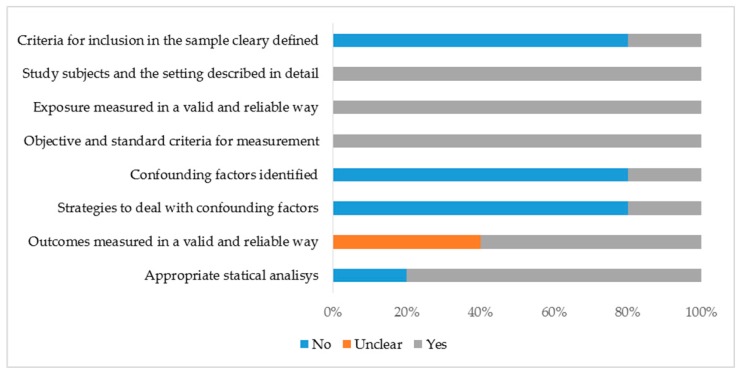
Risk of bias in the included studies (The Joanna Briggs Institute Critical Appraisal Checklist for Analytical Cross-Sectional Studies).

The studies were heterogeneous in relation to a critical appraisal of quality and experimental design and only one had a low risk of bias [13]. Four parameters were completely fulfilled in all studies: (1) study subject and the setting described in detail; (2) exposure measured in a valid and reliable way; (3) objective and standard criteria for measurement; and (4) appropriate statistical analysis used. With regard to outcomes measured, two studies did not clearly define weight regain [10,18]. Other parameters such as inclusion criteria, identification, and strategies to deal with confounding factors were less frequently considered across studies (Figure 2). 

### 3.4. Results of Individual Studies

In two studies [12,13], the majority of patients achieved adequate weight loss, established as more than 50% of excess weight loss, regardless of surgery type. This level of success was reached by 59.8% [12] and 84% [13] of the patients, with mean postoperative time of 24 and 18 months, respectively. Meanwhile, even with adequate excess weight loss, in these studies, the authors observed a significant weight regain after 12 [12] and 24 months [13] post-surgery. The highest prevalence of significant weight regain (47%) was found by Kofman et al. [13]. Moreover, in most studies, a significant correlation between time since surgery and weight regain was observed [12,13,14].

With regard to grazing behavior, the prevalence ranged from 16.6% [12] to 46.6% [13]. As well as weight regain, grazing behavior was also related to time of surgery [12,14]. Conceição et al. [12] observed a grazing prevalence of 16% after 6 months post-operation, and this prevalence reached 45.3% after 2 years. The presence of other eating behaviors such as LOC increased three-fold the risk of reporting grazing [12]. Conceição et al. [10] found association between grazing and weight regain, and also a strong correlation with eating disorder psychopathology (r_sp__0.4; *p* < 0.05). 

Of the five evaluated studies, the association between grazing and weight regain was observed in four [10,12,13,14]. Indeed, Kofman [13] observed that the participants who reported a grazing episode at least two times a week had greater weight regain than those who grazed less frequently (*t =* 6.6, *p* < 0.01). 

## 4. Discussion

In this systematic review, the findings on the association between grazing behavior and weight regain after bariatric surgery were consistent across studies, regardless of surgery type, patient nationalities, and concepts applied to grazing. In general, the repetitive eating behavior, in an unplanned manner not associated with hunger or satiety sensations, might be related to poor quality of life and worse outcomes. These findings indicate that its presence may actually impair the success of the bariatric surgery [12,13,31].

The prevalence of grazing behavior observed in this study varied greatly, from approximately 17 to 50%, and was similar to the values from 18.6 to 59.8%, presented in the first narrative review by Conceição et al. [11]. This high variation may be due to the evaluation moment, since the follow-up time ranged from 6 months [12] to 10 years [13] after surgery, and this eating behavior tends to increase with surgery time [12]. 

The main result of this study was the consistent association between grazing behavior and weight regain post-bariatric surgery, although further studies are warranted to confirm and strengthen this evidence. Only one study did not show this association, but the author suggested that the instrument applied to assess grazing behavior might not have been the ideal tool [18]. Conceição et al. 2017 [10] provide a new screening self-report measure, called Repetitive Eating Questionnaire Rep(eat)-Q, which can optimize the assessment of grazing in bariatric surgery population. Since the presence of different eating patterns such as BED [32,33] or LOC [34] have been linked to weight regain, the presence of grazing was also expected to be a risk factor for this condition. The grazing behavior may emerge or worsen after bariatric surgery once changes in the stomach anatomy require adaptive dietary modifications. Actual guidelines for nutritional treatment of post-bariatric surgery patients endorse eating slowly, chewing food thoroughly, and increasing the frequency of meals [35,36]. In fact, it should be made clear that if eating and choosing food in a controlled manner, mindful of hunger and satiety sensations, this eating pattern cannot be considered as grazing behavior [37]. However, some patients continue to keep eating compulsively, and they shift their eating pattern from bingeing to grazing since they are not able to eat large quantities of food [10,13,32,37]. However, further investigation is needed since Nicolau et al. [14] did not find any difference with regard to energy intake between “grazers” and “no grazers”. Saunders [38] reported that patients with BED before surgery turn to grazing 6 months after surgery. Similarly, Colles et al. [31] found that approximately 60% of preoperative BED patients became grazers 1 year after bariatric surgery and 94% of preoperative grazers continued to report this eating behavior after surgery. In turn, Simpson [18] found a positive correlation between binge eating and grazing behavior in postoperative bariatric patients, suggesting that both eating disorders can occurs simultaneity. Another eating behavior that can predict grazing is the presence of LOC [12]. One of the studies included in this review showed that half of participants reported LOC, and this behavior was also associated to weight regain [13]. In contrast, Conceição et al. [12] did not show any association between LOC and weight regain, although LOC has been a predictor for grazing development [37]. In preoperative patients, grazing has also been diagnosed [31,39].

In addition to eating behavior, hormonal adaptations are observed in bariatric patients and are related to body weight control. It has been suggested that patients with weight regain after bariatric surgery may present an increase in ghrelin concentration and a reduction of the peptideYY, which favors a higher caloric intake [5]. The inability to suppress postprandial ghrelin levels may also be a factor that can explain the development of grazing behavior in obese individuals [40]. Thus, the types of surgery and control of possible confounding factors, such as hormonal imbalance, are important elements for understanding these associations in future studies.

The strengths of this study are the systematic review protocol, which allows the identification of good quality studies; the rigorous assessments of subjects with face-to-face interviews; and the inclusion of studies covering multiple forms of bariatric surgery and postsurgical long-term follow-up, representing a good scope of the studies evaluated.

Some limitations should be considered. There is no unique grazing definition among studies, which may impair the study’s results. Moreover, all of the included studies were of cross-sectional design, limiting the ability to examine causal relationships. 

## 5. Conclusions

There is consistent evidence suggesting that grazing behavior could lead to weight regain in post-surgery bariatric patients, regardless of surgery type and contextual concept of grazing. Further studies are needed to clarifying the real prevalence and interfering factors in order to support the best weight outcomes for bariatric surgery patients.

## Figures and Tables

**Table 1:** Summary of characteristics and outcomes of the included studies.

Author, Publication and Year	Aim of Study	Study Design/Follow-Up Period	Type of Surgery	Sample Size	Grazing Behavior Evaluative Instrument	Weight Regain Cut-Off	Prevalence of Weight Regain (%)	Grazing Behavior Prevalence (%)	Main Result of Grazing and Weight Regain
Conceição et al., 2014	To investigate the different maladaptive	Cross-sectional/6 months to 2 years	RYGB or LAGB	6 months *n* = 90	Bariatric version of the EDE-BSV	EWR > 15%	6 months = no weight regain	6 months = 16.6%	2 years
	eating behaviors at pre- and post-bariatric surgery and weight outcomes			1 year *n* = 96			1 year = 10.4%	1 year = 36.4%	Wald χ^2^(1) = 16.2
				2 years *n* = 117			2 years = 23.2%	2 years = 45.3%	*p* < 0.001, 95% CI −1.1–−0.4
Kofman et al., 2010	To assess the relationship between eating behaviors, weight outcomes and quality of life	Cross-sectional/3 to 10 years	RYGB	497	QEWP-R with 2 questions added about episodes frequency and LOC	Ranged from 0 to >50%, at intervals of 10%	87%	46.6%	*r =* 0.39
									*p* < 0.001
Nicolau et al., 2014	To assess the grazing influence in clinical, biochemical, and psycological outcomes	Cross-sectional/≥18 months	RYGB and sleeve gastrectomy.	60	Structured interview	EWR > 10%	36.7%	41.6%	72% of “grazers” had weight regain
									*p* < 0.001
Simpson, 2016	To evaluate if eating behavior could predict short- and long-term success post-surgery	Cross-sectional/≥12 months	RYGB, LAGB and Sleeve gastrectomy	50	The Grazing Questionnaire (score 0–32)	Not reported.	44% of the sample had weight regain.	Mean score = 10.5 (5.76)	*r* = −0.15
									*p* = 0.33
Conceição et al., 2017	To validate the Rep(eat)-Q and investigate its association with BMI and psychopathology	Cross-sectional/≥12 months;	RYGB or Sleeve gastrectomy	84	Rep(eat)-Q,	Not reported	Not reported	18.0% *	*r* = 0.28
									*p* < 0.001

BMI, Body Mass Index; EDE-BSV, Eating Disorder Examination-Bariatric Surgery Version; EWR, Excess weight regain; LAGB, Laparoscopic Adjustable Gastric Banding; LOC, Loss of control; RYGB, Roux-en-Y Gastric Bypass; SG, Sleeve Gastrectomy; QEWP-R, Questionnaire on Eating and Weight Patterns Revised; Rep(eat)-Q, Repetitive eating questionnaire; * Grazing prevalence in total sample (pre- and post-bariatric surgery).

## Appendix A

**Table A1:** Database search strategies.

Database	Search (19 October 2017)
MEDLINE	(“bariatric surgery” [All Fields] OR (“gastroplasty” [MeSH Terms] OR “gastroplasty” [All Fields]) OR “metabolic surgery” [All Fields] OR “stomach stapling” [All Fields] OR “bariatric surgical procedure” [All Fields] OR bariatric[All Fields] OR “gastric bypass” [All Fields] OR “stomach bypass” [All Fields] OR “Roux in Y” [All Fields] OR “Roux in Y gastric bypass” [All Fields] OR “RYGB” [All Fields] OR “sleeve gastrectomy” [All Fields] OR “obesity surgery” [All Fields] OR “surgery for obesity” [All Fields] OR “gastric banding” [All Fields] OR “gastric band” [All Fields] OR “vertical banded gastroplasty” [All Fields] OR “banded gastroplasty” [All Fields]) AND (graze[All Fields] OR grazing[All Fields] OR picking[All Fields] OR pick[All Fields] OR nibbling[All Fields] OR nibble[All Fields] OR “eating disorders” [All Fields] OR “eating behavior” [All Fields] OR “feeding behavior” [All Fields] OR “feeding disorders” [All Fields] OR “snacking” [All Fields] OR (“snacks” [MeSH Terms] OR “snacks” [All Fields] OR “snack” [All Fields]) OR “eating episode” [All Fields] OR “between-meal snacking” [All Fields]) AND ((“weights and measures” [MeSH Terms] OR (“weights” [All Fields] AND “measures” [All Fields]) OR “weights and measures” [All Fields] OR “weight” [All Fields] OR “body weight” [MeSH Terms] OR (“body” [All Fields] AND “weight” [All Fields]) OR “body weight” [All Fields]) OR “weight gain” [All Fields] OR “weight regain” [All Fields] OR “weight maintenance” [All Fields] OR “weight relapse” [All Fields] OR “weight recidivism” [All Fields] OR “weight loss” [All Fields] OR “weight loss failure” [All Fields])
EMBASE	‘bariatric surgery’ OR gastroplasty OR ‘metabolic surgery’ OR ‘stomach stapling’ OR ‘bariatric surgical procedure’ OR bariatric OR ‘gastric bypass’ OR ‘stomach bypass’ OR ‘roux in y’ OR ‘roux in y gastric bypass’ OR ‘rygb’ OR ‘sleeve gastrectomy’ OR ‘obesity surgery’ OR ‘surgery for obesity’ OR ‘gastric banding’ OR ‘gastric band’ OR ‘vertical banded gastroplasty’ OR ‘banded gastroplasty’ AND (graze OR grazing OR picking OR pick OR nibbling OR nibble OR ‘eating disorders’ OR ‘eating behavior’ OR ‘feeding behavior’ OR ‘feeding disorders’ OR ‘snacking’ OR snack OR ‘eating episode’ OR ‘between-meal snacking’) AND (weight OR ‘weight gain’ OR ‘weight regain’ OR ‘weight maintenance’ OR ‘weight relapse’ OR ‘weight recidivism’ OR ‘weight loss’ OR ‘weight loss failure’)
COCHRANE	‘(“bariatric surgery” OR gastroplasty OR “metabolic surgery” OR “stomach stapling” OR “bariatric surgical procedure” OR bariatric OR “gastric bypass” OR “stomach bypass” OR “Roux in Y” OR “Roux in Y gastric bypass” OR “RYGB” OR “sleeve gastrectomy” OR “obesity surgery” OR “surgery for obesity” OR “gastric banding” OR “gastric band” OR “vertical banded gastroplasty” OR “banded gastroplasty”) in Title, Abstract, Keywords and (graze OR grazing OR picking OR pick OR nibbling OR nibble OR “eating disorders” OR “eating behavior” OR “feeding behavior” OR “feeding disorders” OR “snacking” OR snack OR “eating episode” OR “between-meal snacking”) in Title, Abstract, Keywords and (weight OR “weight gain” OR “weight regain” OR “weight maintenance” OR “weight relapse” OR “weight recidivism” OR “weight loss” OR “weight loss failure”) in Title, Abstract, Keywords in Cochrane Reviews’
LILACS	(tw:((“bariatric surgery” OR gastroplasty OR “metabolic surgery” OR “stomach stapling” OR “bariatric surgical procedure” OR bariatric OR “gastric bypass” OR “stomach bypass” OR “Roux in Y” OR “Roux in Y gastric bypass” OR “RYGB” OR “sleeve gastrectomy” OR “obesity surgery” OR “surgery for obesity” OR “gastric banding” OR “gastric band” OR “vertical banded gastroplasty” OR “banded gastroplasty”))) AND (tw:((graze OR grazing OR picking OR pick OR nibbling OR nibble OR “eating disorders” OR “eating behavior” OR “feeding behavior” OR “feeding disorders” OR “snacking” OR snack OR “eating episode” OR “between-meal snacking”))) AND (tw:((weight OR “weight gain” OR “weight regain” OR “weight maintenance” OR “weight relapse” OR “weight recidivism” OR “weight loss” OR “weight loss failure”)))
SCOPUS	TITLE-ABS-KEY (“bariatric surgery” OR gastroplasty OR “metabolic surgery” OR “stomach stapling” OR “bariatric surgical procedure” OR bariatric OR “gastric bypass” OR “stomach bypass” OR “Roux in Y” OR “Roux in Y gastric bypass” OR “RYGB” OR “sleeve gastrectomy” OR “obesity surgery” OR “surgery for obesity” OR “gastric banding” OR “gastric band” OR “vertical banded gastroplasty” OR “banded gastroplasty”) AND TITLE-ABS-KEY (graze OR grazing OR picking OR pick OR nibbling OR nibble OR “eating disorders” OR “eating behavior” OR “feeding behavior” OR “feeding disorders” OR “snacking” OR snack OR “eating episode” OR “between-meal snacking”) AND TITLE-ABS-KEY (weight OR “weight gain” OR “weight regain” OR “weight maintenance” OR “weight relapse” OR “weight recidivism” OR “weight loss” OR “weight loss failure”) AND (LIMIT-TO (DOCTYPE, “ar”) OR LIMIT-TO (DOCTYPE, “re”) OR LIMIT-TO (DOCTYPE, “ip”) OR LIMIT-TO (DOCTYPE, “sh”))
WEB OF SCIENCE	#1 TS = (“bariatric surgery” OR gastroplasty OR “metabolic surgery” OR “stomach stapling” OR “bariatric surgical procedure” OR bariatric OR “gastric bypass” OR “stomach bypass” OR “Roux in Y” OR “Roux in Y gastric bypass” OR “RYGB” OR “sleeve gastrectomy” OR “obesity surgery” OR “surgery for obesity” OR “gastric banding” OR “gastric band” OR “vertical banded gastroplasty” OR “banded gastroplasty”) AND #2 TS = (graze OR grazing OR picking OR pick OR nibbling OR nibble OR “eating disorders” OR “eating behavior” OR “feeding behavior” OR “feeding disorders” OR “snacking” OR snack OR “eating episode” OR “between-meal snacking”) AND #3 TS = (weight OR “weight gain” OR “weight regain” OR “weight maintenance” OR “weight relapse” OR “weight recidivism” OR “weight loss” OR “weight loss failure”)
GOOGLE SCHOLAR	With all of the words: “bariatric surgery” With at least one of the words: graze OR grazing OR picking OR pick OR nibbling OR nibble OR “eating disorders” OR “eating behavior” OR “feeding behavior” OR “feeding disorders” OR “snacking” OR snack OR “eating episode” OR “between-meal snacking” Where my words occurs: anywhere in the article 200 most relevant hits
PROQUEST	All (“bariatric surgery”) AND grazing AND all (weight)
OPEN GREY	“bariatric surgery” AND grazing AND “weight loss”
